# Coping in Centenarians: Patterns and Correlates

**DOI:** 10.1093/geroni/igab046.417

**Published:** 2021-12-17

**Authors:** Kim Uittenhove, Daniela Jopp, Kathrin Boerner

**Affiliations:** 1 UNIL, Lausanne, Vaud, Switzerland; 2 University of Lausanne, Lausanne, Vaud, Switzerland; 3 University of Massachusetts Boston, Boston, Massachusetts, United States

## Abstract

Coping strategies are a source of resilience, yet little is known about their use in centenarians. We examined patterns in coping strategy use and determined how these patterns were associated with characteristics such as personality, cognitive status, quality of life, and health. We analyzed data from the Fordham Centenarian Study (N = 119), where centenarians responded to 40 items covering 10 coping dimensions (e.g., active problem-solving, support seeking, reappraisal). Findings revealed two clusters which differed in amount and strategy types: One was characterized by high use of many strategies which addressed the problem and its appraisal. The other was characterized by a smaller strategy repertoire, with very limited use of problem-focused strategies. The more varied and problem-focused coping pattern was associated with other characteristics, such as personality (e.g., extraversion) and quality of life (e.g., well-being). Findings suggest variation in coping profiles associated with resilience in centenarians.

